# Effects of Grazing Regimes on Plant Traits and Soil Nutrients in an Alpine Steppe, Northern Tibetan Plateau

**DOI:** 10.1371/journal.pone.0108821

**Published:** 2014-09-30

**Authors:** Jian Sun, Xiaodan Wang, Genwei Cheng, Jianbo Wu, Jiangtao Hong, Shuli Niu

**Affiliations:** 1 Synthesis Research Centre of Chinese Ecosystem Research Network, Key Laboratory of Ecosystem Network Observation and Modelling, Institute of Geographic Sciences and Natural Resources Research, Chinese Academy of Sciences, Beijing, China; 2 The key laboratory of mountain surface processes and eco-regulation, Institute of Mountain Hazard and Environment, Chinese Academy of Sciences, Chengdu, China; Institute of Botany, China

## Abstract

Understanding the impact of grazing intensity on grassland production and soil fertility is of fundamental importance for grassland conservation and management. We thus compared three types of alpine steppe management by studying vegetation traits and soil properties in response to three levels of grazing pressure: permanent grazing (M1), seasonal grazing (M2), and grazing exclusion (M3) in the alpine steppe in Xainza County, Tibetan Plateau. The results showed that community biomass allocation did not support the isometric hypothesis under different grassland management types. Plants in M1 had less aboveground biomass but more belowground biomass in the top soil layer than those in M2 and M3, which was largely due to that root/shoot ratios of dominant plants in M1 were far greater than those in M2 and M3. The interramet distance and the tiller size of the dominant clonal plants were greater in M3 than in M1 and M2, while the resprouting from rhizome buds did not differ significantly among the three greezing regimes. Both soil bulk density and soil available nitrogen in M3 were greater than in M1 at the 15–30 cm soil depth (*P* = 0.05). Soil organic carbon and soil total nitrogen were greater in M3 than in M1 and M2 (*P* = 0.05). We conclude that the isometric hypothesis is not supported in this study and fencing is a helpful grassland management in terms of plant growth and soil nutrient retention in alpine steppe. The extreme cold, scarce precipitation and short growing period may be the causation of the unique plant and soil responses to different management regimes.

## Introduction

Alpine grasslands make up the dominant ecosystem occupying approximately 94% of Northern Tibet [1]. The natural environment of the region is extremely harsh, and the alpine steppe, a fragile ecosystem, is extremely susceptible to the impacts of human activities [2]. It suffers from overgrazing, deforestation, and the harvesting of numerous herbs commonly used in traditional medicines [3]–[5]. Studies examining the response of above- and belowground biomass, the root/shoot ratio, the morphological characteristics of dominant plants, and the soil properties to human disturbance offer important insights that can contribute to adopting the most effective approach to grassland management in an alpine steppe, in which it is particularly difficult to recover from ecosystem degradation due to the region's long period of frost and relatively short growing season [3], [6].

Although grazing and fencing, both of which have a substantial affect on vegetation traits and soil properties [7]–[9], are the most prevalent management regimes for grasslands worldwide, and although the effects of herbivores on soil properties in (sub)alpine ecosystems have recently been reported [10], [11], knowledge about plants and soils in response to grassland management regimes (i.e., fencing and grazing) in the Tibetan Plateau remains limited due to an extremely difficult geographic situation [9], [12]. With regard to soil properties, it has been documented that grazing depresses soil carbon storage by changing the plant biomass and composition of a Tibetan alpine meadow [13]. In contrast, Shi et al. [14] found grazing exclusion to decrease soil organic carbon storage in an alpine grassland of the Tibetan Plateau, while another report suggested that seasonal grazing might enrich soil nutrients [15]. Such conflicting results indicate that different grazing intensities may have varying impacts on soil properties.

Understanding the influence of different management types on grassland production is essential for improving grassland conservation and management [16]. Previous studies have yielded varying results for aboveground biomass changes. Grazing thus increased [17] or decreased [18] aboveground production in different cases. The fencing optimization hypothesis posits that fencing significantly enhances above- and belowground biomass by means of carbon reallocation to plant growth and promotes increases in soil nutrient concentrations [19], [20]. Belowground biomass has also been shown to be affected by grazing, with some studies indicating that plants reduce the proportion of aboveground parts and allocate more biomass to belowground parts, so as to geminate and resist environmental stress (i.e., grazing pressure) [21]. Conversely, more belowground biomass was allocated to the surface layer than the subsurface of the soil profile with increasing grazing pressure [22]. For instance, the root to shoot ratio in the grazing pattern was significantly higher than for the mowing and fencing patterns in temperate grassland [23].

The root∶shoot ratio provides the basis for understanding the response or adaptive strategies of plants to environmental stress [24], [25], and reflects how plants respond to different selection pressures [26], [27]. Theory predicts the ratio of roots to shoots to respond isometrically across different individuals and community types under varying environmental conditions [28]–[30]. While biomass allocation has been widely examined from individual to community to ecosystem levels [31]–[35], few studies compare the biomass partitioning of dominant alpine species under different grassland management types.

Aside from biomass, the morphological characteristics of clonal plants are also regarded as a sensitive index for assessing the possible effect of grazing pressure [36], [37]. The traditional theory is that a clone with a guerrilla-like foraging strategy should be able to locate and exploit favorable patches in which competitors are absent. In a habitat in which potential competitors are more homogeneously distributed, a phalanx clone exhibits a “consolidation” strategy and may be able to persist and monopolize locally available resources [38]. However, we know little about the responses of the morphological characteristics of clonal plants to grazing pressure in alpine steppes.

In this study, we examined plant traits and soil properties in response to different grassland management strategies in the Northern Tibetan Plateau. The specific aims are: (1) to reveal the impact of different grassland managements on plant traits and soil properties and to test the grazing optimization hypothesis; (2) to test the isometric partitioning theory on different alpine species under varying grassland management patterns; and (3) to explore the effects of grazing stress on clonal growth.

## Materials and Methods

### Study area

**Figure 1:**
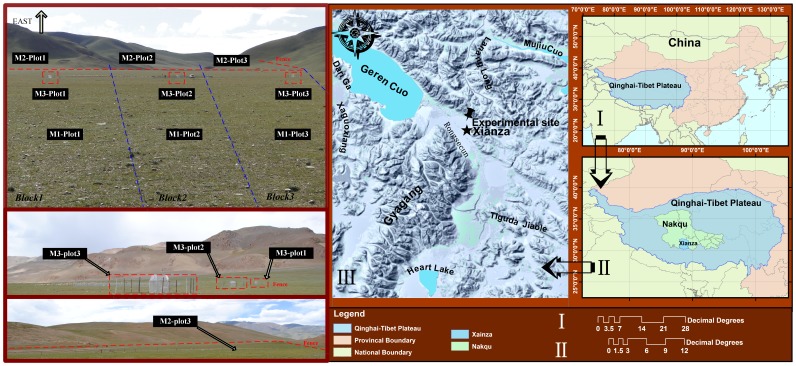
The experiment sites in alpine steppe, northern Tibetan Plateau.

**Figure 2:**
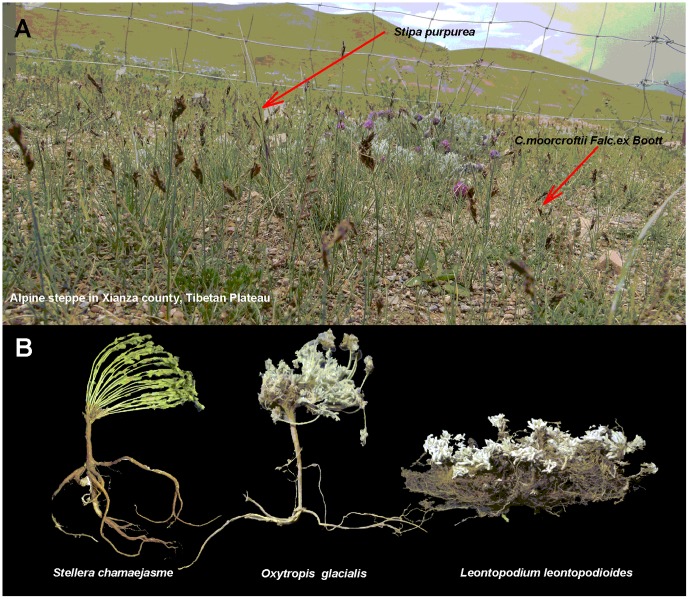
The vegetation composition diagram is in the study area. The Vegetation coverage of the alpine steppe was about 20%, which are dominated by *Stipa purpurea* and *Carex moorcrofii* (panel A) and the accompanying species are *Stellera chamaejasme Linn.*, *Leontopodium alpinum*, *Oxytropis microphylla* (panel B) and so on.

Our study was conducted in the Alpine Steppe Experiment Station (N 30°57′, E 88°42′, 4675 m a.s.l.) in Xainza County, Northern Tibet (Fig. 1). The annual mean air temperature of this region is 0°C, while the mean air temperature ranges from −10.1°C in January and 9.6°C in July. The average annual precipitation is 300 mm. There is no absolute frost-free season, and the frosty period can last up to 279.1 days [39]. The region's soil is classified as steppe soil with sandy loam [40]. Vegetation coverage of the alpine steppe is approximately 20% and is dominated by *Stipa purpurea* (Bunch grass) and *Carex moorcrofii* (sedge family, creeping rhizomes) (Fig. 2A). Other common species include *Stellera chamaejasme Linn.* (Bunch weeds), *Oxytropis glacialis* (Bunch weeds), and *Leontopodium alpinum* (Fig. 2B). All of these plants are perennial herbs.

### Grazing treatments and experimental plots

There were three grazing treatments in this study. i.e, M1 (grazing): The pasture is always used for grazing. M2 (seasonal grazing): The pasture has been used for grazing in the non-growing seasons but not in the growing season (from May to September) since 2007. M3 (grazing exclusion): Herbivores have been excluded from the pasture since 2010.

The study area was divided into three blocks. Each block contained three plots, the eastern plot having a grazing intensity of M2, the middle plot a grazing intensity of M3, and the western plot a grazing intensity of M1 (Fig. 1). The dimensions of each M3 fenced plot were 6×6 m; the dimensions for each M2 plot were 200×500 m. Three M1 plots were located outside of the fenced area in each block, with each plot having dimensions of 200×500 m. In each plot, a quadrat (30 cm×30 cm) was used to measure biomass and take samples. The estimated average livestock density in both M1 and M2 was approximately 120 sheep units per km^2^. (The “sheep unit” is the most frequently used unit of measurement for evaluating the carrying capacity of pasture areas in China and includes figures for cattle, which are converted to sheep; thus, e.g., one cow is equivalent to five sheep based on the average weight of a cow) [41].

### Ethics statement

No specific permits were required for the samples collected from any of the sites, and the field studies did not involve endangered or protected species.

### Biomass measure and sample taking

We established a 30 cm×30 cm quadrat in each of the sampling plots to harvest aboveground biomass (AGB) in late August of 2012. After harvesting the aboveground biomass, we took soil samples by auger with a 5-cm core diameter at two depths (0–15 cm and 15–30 cm) to estimate belowground biomass (BGB). According to previous research, most belowground biomass is located in the top 30 cm layer [42]. Soil samples were also taken at depths of 0–15 cm and 15–30 cm in order to measure soil elements. After being air-dried and sieved (2 mm mesh), the soil samples were carefully handpicked to extract the surface organic materials and fine roots for an analysis of soil chemical properties. Soil nutrients (including soil organic carbon, soil total nitrogen content, available nitrogen, total phosphorus and available phosphorus) were determined using standard protocol [43].

In order to estimate the root∶shoot ratio of the dominant species (*S. purpurea*, *C. moorcrofii*, *O. glacialis*, and *L. alpinum*), we took three soil blocks in each sampling plot of different management regimes. The patch was excavated with a spade with a diameter of 20 cm to a depth of 30 cm, which was selected based on the root morphological traits of the species [42]. The root samples obtained from the sites were immediately placed in a cloth bag and then soaked in water to remove the residual soil by means of a 0.5 mm sieve. The roots of each sample were carefully separated from soil and other belowground materials first. Then individuals of the all species were carefully separated from other plant roots. Finally, we separated the roots and shoots of each individual. Individual biomass (shoots and roots) and community biomass (AGB and BGB) was oven-dried at 65°C until a constant weight was achieved. Individual biomass data were used to analyze allometric functions under different management regimes (with a total of 40, 45, and 68 individuals in M1, M2, and M3, respectively).

The plant morphology of *C. moorcrofii* is regarded as a fairly good indicator that reflects the different grassland management regimes [44]. We thus sampled *C. moorcrofii*. from other 30 cm×30 cm quadrats at each of the 9 plots. The samples were then taken back to the laboratory, where the morphological characteristics (plant height, interramet distance, number of rhizome buds, and tillering number) were immediately measured. Special attention was paid to avoid destroying the structural integrity of the plant throughout this process.

### Statistical analysis

We performed a standardized major axis (SRMA) regression to examine whether AGB and BGB scale isometrically at an individual level across all samples, and then explored their relationships under different management types. The regression relationship of the form *LogAGB* = *α*+*βLogBGB* was used to describe the allometric relationship between AGB and BGB, where x is BGB, y is AGB, *α* is the intercept, and *β* is the scaling slope [25], [28], [45], [46]. The scaling slope and y-intercept of the allometric function were determined using the software package titled “Standardized Major Axis Tests & Routines Version 2.0” [47]. If a 95% confidence interval of the scaling slope covered 1.0, the relationship between AGB and BGB was considered to be isometric.

One-way ANOVA was used to test differences in soil properties and biomes (AGB, BGB, and R/S) among the different treatments, and the Tukey test was used to distinguish differences at a *P* = 0.05 level.

## Results

### Biomass partitioning

**Figure 3:**
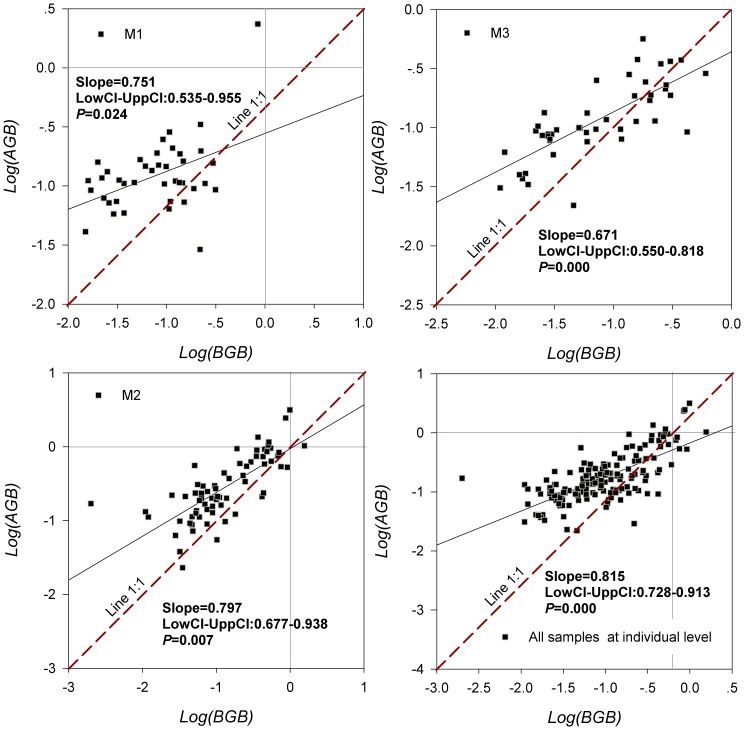
Relationships between AGB and BGB under grazing (M1), seasonal grazing (M2), and grazing exclusion (M3) regimes.

The relationship between BGB and AGB under different grazing pressures was characterized by the linear function *LogAGB* = *α*+*βLogBGB* (Fig. 3). The slopes of the allometric relationship were 0.751 (for M1), 0.797 (for M2), 0.671 (for M3), and 0.815 (for all samples at an individual level), respectively. All of the slopes differed significantly from 1 at *P* = 0.05.

### Responses of AGB, BGB, and R/S to different management types

AGB in M1 was slightly but not significantly lower than in M2 and M3 (Table 1). In contrast, BGB of M1, M2, and M3 amounted to 324.87, 244.84, and 261.88 g·m^−2^, respectively. The ratio of root to shoot in M1 was also higher than in M2 and M3. The root biomass was mainly distributed in a soil depth of 0–15 cm, while the root biomass in a soil depth of 15–30 cm only accounted for 2.77–5.62% of total BGB. The ratios of BGB in the 0–15 cm-layer to BGB in the 15–30 cm-layer of M1, M2, and M3 were 26.19, 35.17, and 16.79, respectively.

**Table 1:** Aboveground biomass (AGB), belowground biomass (BGB), and their ratios (R/S) under different grassland managements.

Management Types	Category	M1		M2		M3	
		Mean	STDEV	Mean	STDEV	Mean	STDEV
**AGB (g·cm^−2^)**		48.09	12.39	51.09	35.48	62.25	3.21
	**0–15 cm**	312.92	65.6	238.07	53.95	247.16	38.96
**BGB (g·cm^−2^)**	**15–30 cm**	11.95	7.64	6.77	5.64	14.72	10.27
	**Ratio(0–15 cm/15–30 cm)**	26.19	8.59	35.17	9.57	16.79	3.79
	**0–30 cm**	324.87	58.18	244.84	59.59	261.88	48.67
**R/S**		7.37	3.58	5.79	2.23	4.2	0.67

**Note:** The differences of biomes under the different grassland management type were insignificant at 0.05 level.

**Figure 4:**
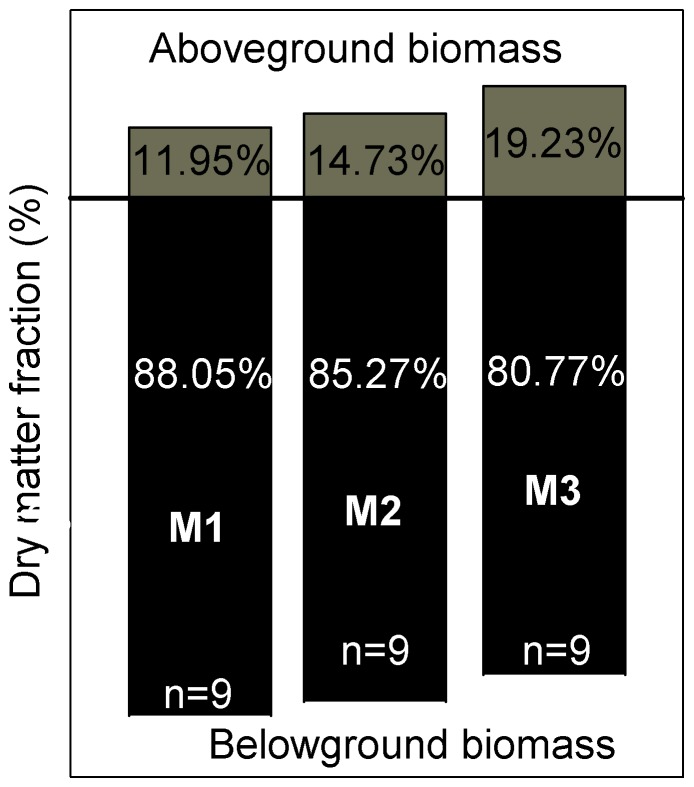
Biomass fractions (% of total biomass) in herbaceous plants in M1, M2 and M3 of Tibetan Plateau.

To investigate the characteristics of biomass allocation further, we analyzed the dry matter fraction of M1, M2, and M3 in the Tibetan Plateau (Fig. 4). The results indicated that the roots of total plants in the Tibetan Plateau amounted to 88.05% (in M1), 85.27% (in M2), and 80.77% (in M3).

### Traits of clonal plants under different management types

**Figure 5:**
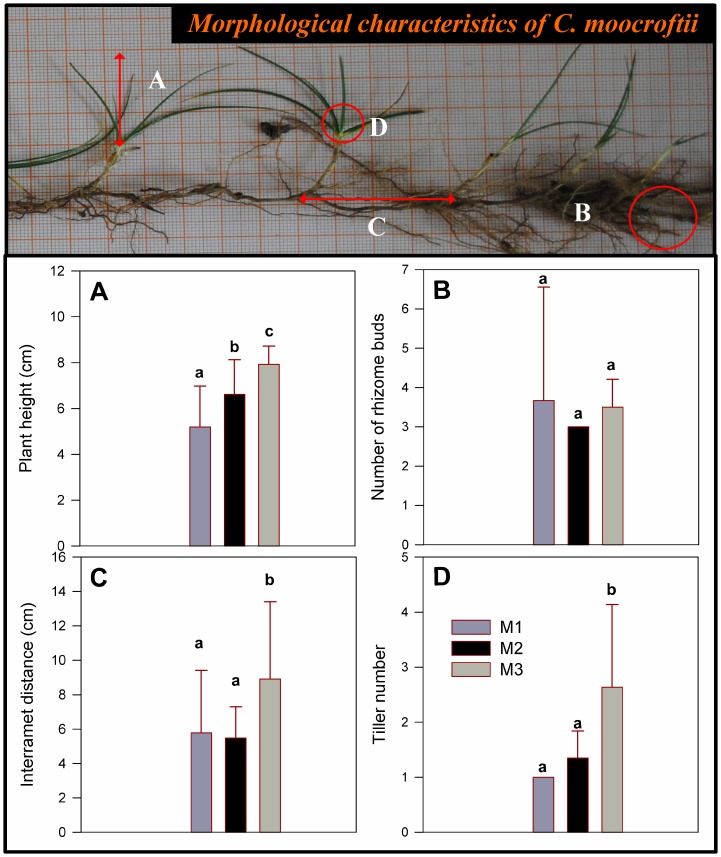
The morphological characteristics of *C. moocroftii* in the different grassland management types. A, represent plant height, B, number of rhizome buds, C, interramet distance, and D tiller number.

*C. moocroftii* was tallest in M3 and shortest in M1 (*P*<0.05, Fig. 5A). Both interramet distance (Fig. 5C) and the tiller number (Fig. 5D) in M3 were significantly larger than in M1 and M2. No significant differences were found in the number of rhizome buds among M1, M2, and M3 treatments (*P*<0.05, Fig. 5B).

### Soil properties in response to management types

At a soil depth of 0–15 cm, soil bulk density, soil organic carbon, total nitrogen, and available nitrogen were non-significant among M1, M2, and M3 (Table 2). At a soil depth of 15–30 cm, both soil bulk densiy and soil available nitrogen in M3 were greater than in M1 at a *P* = 0.05 level. Soil organic carbon and soil total nitrogen in M3 were greater than in M1, and M2 (*P* = 0.05).

**Table 2:** Soil properties under the different grassland managements.

Management Types	Depth	M1		M2		M3	
		Mean	STDEV	Mean	STDEV	Mean	STDEV
**Soil bulk density (g·cm^−3^)**	**0–15 cm**	1.71	0.09	1.48	0.08	1.60	0.11
**Soil organic carbon (g·kg^−1^)**		10.09	3.38	12.72	2.45	11.02	0.85
**Soil total nitrogen(g·kg^−1^)**		1.12	0.26	1.54	0.40	1.19	0.11
**Soil available Nitrogen(mg·kg^−1^)**		62.96	24.14	84.65	17.24	81.61	7.14
**Soil total phosphorous(g·kg^−1^)**		0.34	0.03	0.33	0.04	0.28	0.02
**Soil available phosphorous(mg·kg^−1^)**		3.91	0.72	3.83	0.16	3.39	0.29
**Soil bulk density(g·cm^−3^)**	**15–30 cm**	1.76 **a**	0.04	1.58 **ab**	0.09	1.48 **b**	0.14
**Soil organic carbon (g·kg^−1^)**		5.21 **a**	0.87	5.81 **a**	0.25	10.16 **b**	2.58
**Soil total nitrogen(g·kg^−1^)**		0.64 **a**	0.13	0.57 **a**	0.04	1.07 **b**	0.27
**Soil available Nitrogen(mg·kg^−1^)**		32.36 **a**	4.87	40.57 **ab**	8.51	68.01 **b**	17.37
**Soil total phosphorous(g·kg^−1^)**		0.27	0.01	0.24	0.04	0.37	0.09
**Soil available phosphorous(mg·kg^−1^)**		2.95	0.54	3.25	0.61	3.33	0.11

**Note:** The bold lower case represents the result of variance analysis at 0.05 level.

## Discussion

### Biomass partitioning

Based on the results of our SMA analysis, biomass allocation at the community level did not agree with the isometric hypothesis under different grassland management types. Plants consistently sense changes in their environment and often allocate a greater proportion of their biomass to the root system when water or mineral elements are scarce [48]. Moreover, roots have also been found to store carbohydrates in alpine grasslands [49]. In the alpine steppe, greater plant biomass allocation to belowground reflects the plants' response to the harsh alpine environment (limited precipitation and low temperatures) for survival. Another reason may be the relatively narrow variation in plant size in that region. Moreover, many species in the Tibetan alpine grasslands do not have typical hierarchical branching structures [42], which did not meet the assumption that stem length scales isometrically with respect to root length. This characteristic is clearly reflected in the root morphology structure shown in Fig. 2B and Fig. 5. Biomass allocation in these areas may thus support the allometric biomass partitioning hypothesis, rather than the isometric allocation hypothesis.

### Responses of AGB, BGB, and R/S to different management types

AGB in the grazing plots was found to be lower than in the non-grazing plots, which is in agreement with the findings of Medina-Roldan et al. [50]. We suggested that alpine grassland production might be decreased to some degree by grazing as a result of the consumption effect of the livestock. The higher BGB in M1 than M2 and M3 in our alpine steppe conflict with the results of Cheng et al. [51], who found grazing to significantly decrease belowground biomass in the Loess Plateau. Gao et al. [52] also reported significantly less belowground biomass under conditions of heavy grazing in comparison with a non-grazing site in Inner Mongolia. The conflicting results between our study and previous studies in other areas probably stem from differences in environmental conditions. In the extreme cold region of alpine grassland, plants reduce the proportion of aboveground parts and allocate larger amounts of biomass to belowground parts in order to germinate and resist grazing pressures [21]. More BGB in the top soil layer of M1 than M2 and M3 suggests that grazing results in more belowground biomass being allocated to the topsoil [53], possibly due to the alpine environment inhibiting root growth in ungrazed plots. We did not find an ameliorating effect of reduced grazing (M2) or grazer exclusion (M3), suggesting that the effects of grazing cessation may only be detectable in the long term, as proposed by Steffens et al. [54].

The root∶shoot ratio (R/S) in M1 was much larger than in M2 and M3, suggesting that plants allocate more to BGB than AGB in order to maximize resources for optimal growth in the grazed environment [21]. We compared the dry matter fraction of M1, M2, and M3 in the Tibetan Plateau (Fig. 4) with dry matter fractions in Argentina, Bolivia, Ecuador, the Arctic, and the Alps [49], and we found that the proportion of BGB was much higher in our study sites than for plants in a semi-arid grassland ecosystem (Bolivia), mountain grassland ecosystem (Argentina), and a humid mountain grassland ecosystem (Ecuadorian Andes). The main reason may be associated with the comparatively slow depletion of carbohydrates in roots, resulting from low respiration rates in the extremely cold winter, and the slower root turnover in colder environments [55]. Meanwhile, long-term grazing results in a higher BGB fraction in the Tibetan Plateau than for plants in other cold alpine environments and the Arctic.

### The clonal plant in response to different management types

Clonal plants defend against large herbivores by regulating their morphological characteristics [56]. Clonal plant *C. moocroftii* in M3 was notably taller than in M1 and M2 because it was not bitten by livestock, and they can plastically respond to environmental heterogeneity by placing ramets and changing tiller size in favorable sites [57], [58]. We found the interramet distance and tiller number in M3 to be greater than for plants in M1 and M2. Tillers may produce more daughter tillers as a result of increased nutrient availability in grazing exclusion. *C. moocroftii* thus responds to grazing by decreasing the tiller number [37]. The results also indicate that *C. moocroftii* may have both phalanx and guerrilla strategies. The phalanx strategy involves the production of a compact structure of closely spaced ramets (i.e., M1), and the guerrilla strategy involves the production of loosely arranged and more widely spaced ramets in order to seek out more soil nutrients in a relatively suitable habitat (i.e., M3).

Clonal growth by means of lateral roots is regarded as a typical trait of opportunistic species growing in disturbed habitats, as such growth potentially produces a large number of buds on lateral roots [59]. However, our results implied that different grassland management systems did not affect resprouting from rhizome buds, which suggests that reprouting from rhizome buds is not a major adjustment stragegy in response to grazing intensity. This finding is probably due to the short time period since the implementation of fencing and seasonal grazing.

### Soil properties in response to different management types

Grazing intensity is one of the most important factors influencing soil properties [50], [60]. It has been documented that the trampling action of grazing animals impacts the soil by increasing bulk density [61] and mechanical resistance, and reducing porosity, water infiltration, and aggregate stability [62], and native perennial cover and litter cover, with the consequence of changing the soil nutrient concentrations [63]. In our study, bulk density was found to increase with increasing grazing intensity, and all soil organic carbon, total nitrogen, and available nitrogen contents in M3 were slightly higher than in M1 and M2. We thus report that grazing exclusion may improve soil N availability. Arevalo et al. [64] found an increase in soil phosphorus content in response to increased goat grazing pressure in pastures. In contrast, our results indicated that soil total phosphorous and available phosphorous contents in M1 did not differ significantly from those of M2 and M3. The extreme cold, limited precipitation and short growing period may lead to no obvious change in phosphorus content.

## Conclusions

By studying plant traits and soil nutrients in response to grazing intensity in an alpine steppe, we found that the isometric hypothesis is not supported in this particular region. The extreme cold, limited amount of precipitation and short growing period may lead to the plants' and soils' unique to different management types. Overall, the study suggests that the implementation of grazing exclusion played a positive role in the sustainable development of the alpine steppe region.
